# Supplementary material for: Ni-Catalyzed Enantioselective Intramolecular Mizoroki–Heck Reaction for the Synthesis of Phenanthridinone Derivatives
Source: J Org Chem. 2023 Jun 15;88(13):8203–26. doi: 10.1021/acs.joc.3c00202 (PMC10337041; doi:10.1021/acs.joc.3c00202)
Supplement: Supplementary file 2 — jo3c00202_si_002.zip [file jo3c00202_si_002.zip › Amides (N-MOM)/1i iPr-NMOM-4F-ArBr/19F iPr-NMOM-4F-ArBr 1i/8/pdata/1/email_DR-112-077 F19-22 F19_8_1.pdf]

DR-112-077 F19-22 F19
F19 DMSO {D:\nmrusers\malachowski} BM 2

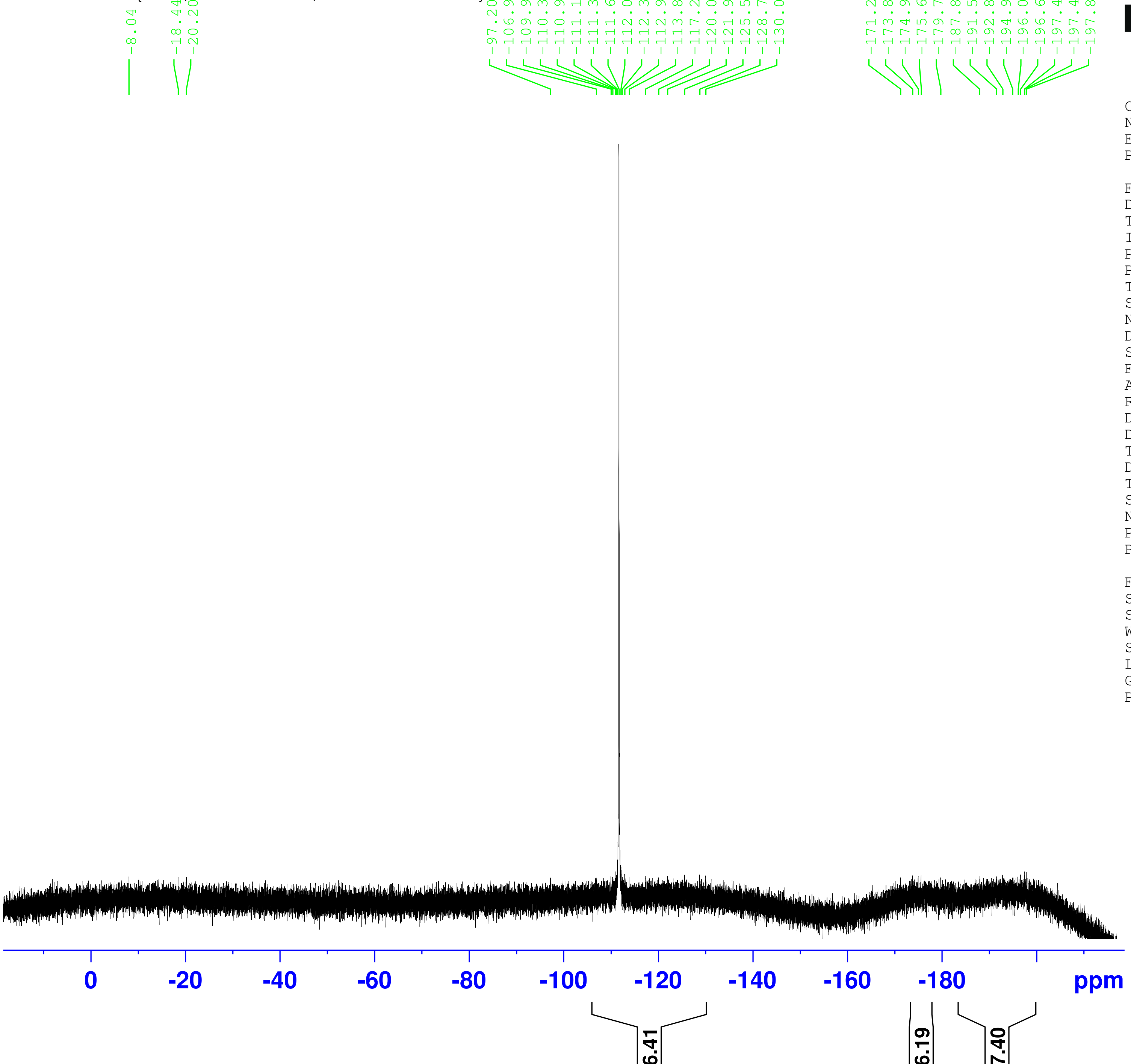

BRUKER

Current Data Parameters

NAME	DR-112-077 F19-22 F19
EXPNO	8
PROCNO	1

F2 - Acquisition Parameters

Date_	20211015
Time	9.39 h
INSTRUM	spect
PROBHD	Z104450_0352 (
PULPROG	zgflqn
TD	131072
SOLVENT	DMSO
NS	16
DS	4
SWH	89285.711 Hz
FIDRES	1.362392 Hz
AQ	0.7340032 sec
RG	203
DW	5.600 usec
DE	6.50 usec
TE	298.0 K
D1	1.00000000 sec
TD0	1
SFO1	376.4795333 MHz
NUC1	19F
P1	12.30 usec
PLW1	19.19499969 W

F2 - Processing parameters

SI	65536
SF	376.5171850 MHz
WDW	EM
SSB	0
LB	0.30 Hz
GB	0
PC	1.00
